# *In-Silico *docking of HIV-1 integrase inhibitors reveals a novel drug type acting on an enzyme/DNA reaction intermediate

**DOI:** 10.1186/1742-4690-4-21

**Published:** 2007-03-20

**Authors:** Andrea Savarino

**Affiliations:** 1 Department of Infectious, Parasitic and Immune-mediated Diseases, Istituto Superiore di Sanità, Viale Regina Elena, 299 00161, Rome, Italy

## Abstract

**Background:**

HIV-1 integrase (IN) is an emerging drug target, as IN strand transfer inhibitors (INSTIs) are proving potent antiretroviral agents in clinical trials. One credible theory sees INSTIs as docking at the cellular (acceptor) DNA-binding site after IN forms a transitional complex with viral (donor) DNA. However, mapping of the DNA and INSTI binding sites within the IN catalytic core domain (CCD) has been uncertain.

**Methods:**

Structural superimpositions were conducted using the SWISS PDB and Cn3D free software. Docking simulations of INSTIs were run by a widely validated genetic algorithm (GOLD).

**Results:**

Structural superimpositions suggested that a two-metal model for HIV-1 IN CCD in complex with small molecule, 1-(5-chloroindol-3-yl)-3-(tetrazoyl)-1,3-propandione-ene (5CITEP) could be used as a surrogate for an IN/viral DNA complex, because it allowed replication of contacts documented biochemically in viral DNA/IN complexes or displayed by a crystal structure of the IN-related enzyme Tn5 transposase in complex with transposable DNA. Docking simulations showed that the fitness of different compounds for the catalytic cavity of the IN/5CITEP complex significantly (*P *< 0.01) correlated with their 50% inhibitory concentrations (IC_50_s) in strand transfer assays *in vitro*. The amino acids involved in inhibitor binding matched those involved in drug resistance. Both metal binding and occupation of the putative viral DNA binding site by 5CITEP appeared to be important for optimal drug/ligand interactions. The docking site of INSTIs appeared to overlap with a putative acceptor DNA binding region adjacent to but distinct from the putative donor DNA binding site, and homologous to the nucleic acid binding site of RNAse H. Of note, some INSTIs such as 4,5-dihydroxypyrimidine carboxamides/*N*-Alkyl-5-hydroxypyrimidinone carboxamides, a highly promising drug class including raltegravir/MK-0518 (now in clinical trials), displayed interactions with IN reminiscent of those displayed by fungal molecules from *Fusarium sp*., shown in the 1990s to inhibit HIV-1 integration.

**Conclusion:**

The 3D model presented here supports the idea that INSTIs dock at the putative acceptor DNA-binding site in a IN/viral DNA complex. This mechanism of enzyme inhibition, likely to be exploited by some natural products, might disclose future strategies for inhibition of nucleic acid-manipulating enzymes.

## Background

Inhibitors of the human immunodeficiency virus type 1 (HIV-1) integrase (IN) enzyme, represent a major advancement in AIDS research, showing potent antiretroviral effects in advanced clinical trials [1-4]. However, despite the decade-long studies in this field (reviewed in: [2]), several questions on the interactions of IN with its inhibitors have remained unanswered [1,2]. These include: the docking site, possible interactions with metal ions and viral DNA, the amino acids involved in binding, the role of drug resistance mutations, and the conformations assumed by the inhibitors in complex with the enzyme. Elucidation of these issues is crucial, given the strict requirement of IN for insertion of proviral DNA into the cell genome, leading to retroviral latency and persistence during therapy [5].

**Figure 1:**
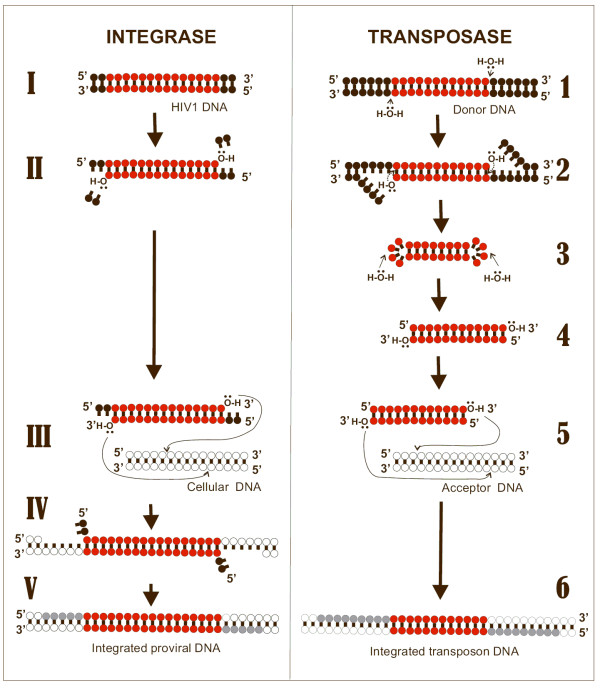
**Sequence of events in HIV-1 integration (left) and Tn5 transposition (right)**. HIV-1: I) donor DNA; II) integrase-catalyzed 3' processing; III) integrase-catalyzed strand transfer; IV) product of strand transfer; V) DNA repair by cellular enzymes. Tn5 transposon: 1) donor DNA; 2) 3'processing; 3–4) 5' processing, consisting of loop formation (3) and generation of blunt-ended DNA (4); 5) strand transfer; 6) repaired strand transfer product. Portions of the donor DNA that become integrated are shown in red. Acceptor DNA is shown in white. Portions of acceptor DNA repaired following the strand transfer reaction are shown in grey.

IN belongs to a family of polynucleotidyl transferases/esterases, comprising transposases, RNAses H, and the Argonaut RNAse associated with Dicer (involved in the gene-silencing pathway) [1,6]. These proteins display similar 3D folding of the catalytic domain and a conserved catalytic triad of metal-coordinating carboxylates. IN catalyses at least two reactions: 1) 3' processing, and 2) strand transfer [reviewed in: [1]] (Fig. 1). Briefly, once the viral RNA is retrotranscribed into DNA by reverse transcriptase, IN-catalyzed 3'-processing removes a 3' terminal portion (usually a dinucleotide) at both ends of HIV-1 DNA (also referred to as donor DNA) (Fig. 1). After 3' processing, IN multimers remain bound to both ends of HIV-1 DNA, and these multimolecular structures, namely pre-integration complexes, translocate to the nucleus [7]. The second reaction catalyzed by IN, *i.e*. strand transfer, inserts both 3' ends of HIV-1 DNA into a host-cell chromosome (referred to as target DNA or acceptor DNA). Strand transfer leaves a five-base, single-stranded gap at each junction between the integrated proviral DNA and the host acceptor DNA, and a (usually) two-base flap at the 5'-ends of the proviral DNA (Fig. 1). The newly formed DNA molecule thus requires repair, likely in coordination with cellular DNA repair enzymes [1]. The lack of 5' cleavage before strand transfer is a major difference between HIV-1 IN and transposases such as Tn5, Tn7 and Tn10, which release a blunt-end transposable element from donor DNA [8,9]. 5' strand cleavage has been shown for Tn5 and Tn10 transposons to occur via a two-step process whereby the 3' OH generated from the initial strand cleavage attacks the 5' strand to form a hairpin, followed by cleavage of the hairpin by attack from an activated water molecule [8,9] (Fig. 1).

**Figure 2:**
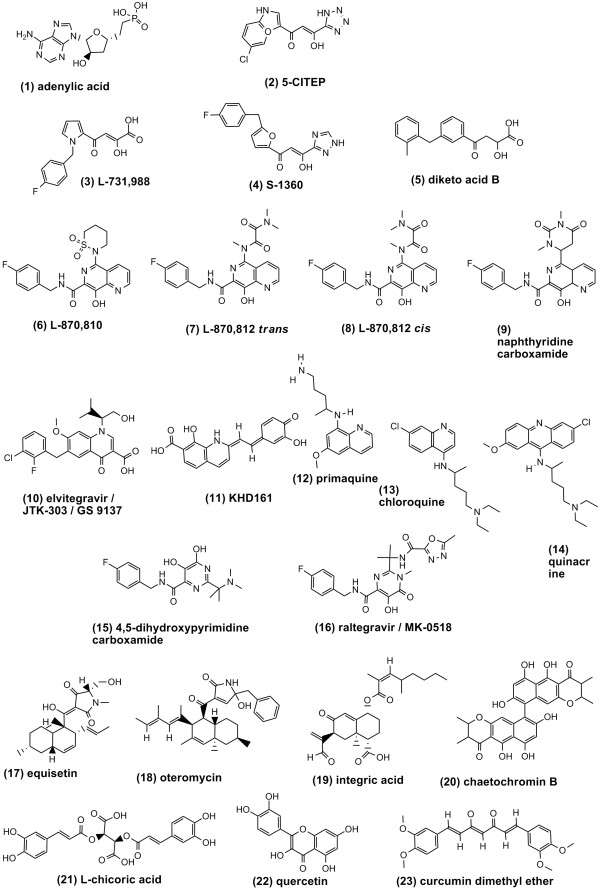
**Compounds mentioned in the present study**. Note that the structure of 8-hydroxy-1,6-naphthyridine carboxamide, L-870,810 is presented both in *trans *and *cis *forms (the latter also referred to L-870,8125) as described in Refs [37] and [31], respectively. The structure of raltegravir/MK-0518 was retrieved from Ref. [54]. All other structures are available in the NCBI website [45].

IN inhibitors can be divided into dual inhibitors of 3' processing and stand transfer (simply referred to as 3'P inhibitors), and selective strand transfer inhibitors (INSTIs). A credible theory sees 3'P inhibitors as docking at the HIV-1 DNA-binding site, and INSTIs as occupying the position of acceptor DNA [1,10]. This theory is supported by biochemical evidence [10,11]. IN inhibitors currently in clinical trials belong to the INSTI group. Chemically, they display a β-hydroxy carbonyl (Fig. 2), thought to bind the (possibly) two metal ions coordinating the three catalytic residues D64, D116 and E152 [2,12]. A crystal structure of these novel antiretrovirals in an IN/DNA complex is still far from being available, and full understanding of the binding mode of these inhibitors has been hampered by lack of information on some important points. These include: 1) a three-dimensional (3D) structure of the catalytic core domain (CCD) presenting both metal ions displayed by structurally related enzymes [8,12-14] (only the metal between D64 and D116 is present in some structures [15,16]); 2) the mobility of a flexible loop in the CCD (residues 140–152, partially or totally absent in most crystal structures and displaying varying configurations when present in its entirety) [17]; 3) crystallographic data on the IN/DNA interaction; 4) a crystal structure of full-length IN (the three domains, N-terminal, C-terminal, and CCD have been solved separately, and their 3D folding in a catalytic complex is only hypothetical) [1]. One crystal structure of Goldgur *et al*. showed 1-(5-chloroindol-3-yl)-3-(tetrazoyl)-1,3-propandione-ene (5CITEP), *i.e*. compound (**2**) (see Fig. 2), lying between the three catalytic residues [16]. Unfortunately, this structure displayed only one of the two possible metal ions within the catalytic cavity. Although some attributed the position of 5CITEP to physical entrapment during crystallization (crystal packing), recent biochemical data confirmed some of the contacts observed by Goldgur *et al*. [10], but showed that 5CITEP, though presenting some structural features of INSTIs, resembles more a 3'P inhibitor [10], in line with enzyme inhibition data in the presence of Mg^++ ^(*i.e*. the metal thought to act as a cofactor *in vivo*) [18].

Given the increasing importance of selective INSTIs for AIDS medicine and their novel mechanism acting upon a protein/DNA complex, some two-metal IN models were created by molecular modeling in an attempt to describe inhibitor binding *in silico *[12,14]. However, the only docking study using a protein/DNA complex was conducted by Barreca *et al*. [19] As a surrogate platform, these authors employed a 3D structure of Tn5 transposase in complex with two metal ions and donor DNA. Other models are however necessary, since susceptibility of HIV-1 IN to INSTIs may be affected by few amino acid changes, as shown by drug-resistance mutation studies [12]. Moreover, the available structures present the Tn5 enzyme in complex with the blunt-end reaction intermediate which is not produced by HIV-1 IN [20]. On the other hand, theoretical structures of the HIV-1 IN in complex with donor DNA (obtained by molecular modelling and *in-silico *automated docking) [21,22] can only hazardously be used as a platform to study inhibitor binding, in the absence of further validation. *In-silico *docking of INSTIs at these models would be the final step of a number of computational simulations (*e.g*. reconstruction of full-length IN, protein/DNA docking), thus harboring the risk of becoming extremely artificial. In the absence of suitable 3D models, reliable information on the interactions of IN with DNA and specific inhibitors is derived from cross-linking experiments [10]. These studies, however, detected only few protein/DNA contacts and cannot furnish a full 3D view of the complex.

Using a 3D platform exploiting crystallographic data on IN CCD in complex with 5CITEP as a surrogate model for *in-silico *docking simulations of INSTIs, the present study provides a first view into an IN active site harbouring the new antiretrovirals. The computational procedures adopted here bypass artificial steps such as *in-silico *reconstruction of full-length IN and IN/DNA complexes, and are limited to one small-molecule inhibitor docking step, using a widely validated genetic algorithm. The docking solutions are in agreement with robust biochemical data in the literature and may disclose new insights into inhibition of an enzyme/substrate reaction intermediate.

## Results and discussion

### The Tn5 transposase/transposable DNA complex shows similarities with and differences from the HIV-1 IN/viral DNA interaction

**Figure 3:**
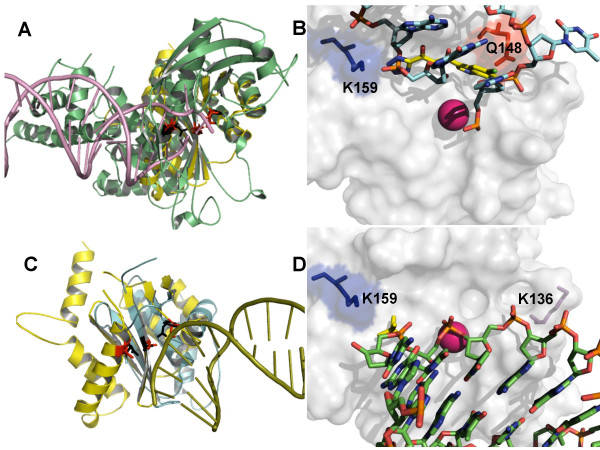
**Mapping of the nucleic acid-binding sites within the HIV-1 integrase (IN) catalytic site**. Panel A: Structural superimposition between the crystal structures of HIV-1 IN catalytic core domain (PDB accession code: 1QS4; in yellow) and Tn5 transposase in complex with donor DNA (PDB: 1MM8; protein in green; DNA: in violet). The catalytic triads of IN and Tn5 transposase are shown in red and black, respectively. Panel B: transposition of Tn5 donor DNA (carbon backbone in cyan) to a crystal structure (PDB:1QS4) of HIV-1 IN in complex with 1-(5-chloroindol-3-yl)-3-(tetrazoyl)-1,3-propandione-ene (5CITEP; carbon backbone in yellow). HIV-1 DNA-interacting residues Q148 and K159 are shown as sticks. Hydrogens have been removed for better clarity. The metal ion crystallized with IN is shown in magenta. Panel C: Structural superimposition between the crystal structures of HIV-1 IN catalytic core domain (in yellow), and *Bacillus halodurans *RNAse H in complex with an RNA/DNA hybrid (PDB: 1ZBL; protein: in cyan; nucleic acid: in smudge green). The catalytic triads of IN and RNAse H are shown in red and black, respectively. Panel D: Transposition of *B. halodurans *RNA/DNA hybrid (carbon backbone in green) to HIV-1 IN. Putative DNA-interacting residues, K136 and K159 (from right to left) are shown as sticks. A phosphate (in yellow) co crystallized with IN (PDB: 2B4J) had been added by superimposing the 2B4J structure to the IN structure (PDB: 1QS4) used as reference structure in this part of the present study.

To map the donor DNA-binding site within the catalytic site of IN, previous work used the crystal structure of inhibitor 5CITEP in complex with HIV-1 IN CCD described by Goldgur *et al*. [22], or a structure of Tn5 transposase in complex with transposable DNA (corresponding to proviral DNA) [19]. To compare these approaches, a structural alignment of the two enzymes was performed in the present study. The alignment involved 75 amino acids including those facing the catalytic cavity. The root mean square deviation (RMSD) was 0.17 Å between the α-carbons of the highly conserved catalytic triads (IN: D64, D116 and E152; transposase: D97, D188 and E326) (Fig. 3A). The 3D similarities between HIV-1 IN and transposases have been extensively described in the literature [for a review, see: [6]].

When the Tn5 DNA was transposed onto the HIV-1 IN CCD structure, a close contact was observed between K159 and the phosphate immediately 5' to the 3' terminal nucleotide (Fig. 3B). One similar contact was described to occur with the phosphate immediately 5' to the 3' processing site of HIV-1 DNA [23], thus supporting the hypothesis that the 3' terminal portions of transposable DNA and HIV-1 3'processed (3'P)DNA occupy similar positions within the active sites of the two enzymes. This hypothesis is further supported by the overlap of the tetrazole ring of 5CITEP (a bioisoestere of the carboxylate anion) with the phosphate contacting K159 (Fig. 3B). Although Tn5 DNA and HIV-1 3'PDNA likely map to corresponding portions of the active sites of the two enzymes, transposable DNA *per se *cannot mimic HIV-1 DNA, because it is a blunt-end reaction intermediate which is not generated in the reactions catalyzed by HIV-1 IN (see Fig. 1). Moreover, the experimental data clearly reveal a loop-like structure at the 5' terminus, a likely product of 5' processing (Fig. 3A–B). Given these reasons, 5CITEP was, in the present study, preferred over transposable DNA as an HIV-1 DNA mimic. In line with this choice, a recent study [10] showed that the contact of Q148 (in the flexible loop) with 5CITEP, displayed by the crystal structure of Goldgur *et al*. [16], was reproducible in cross-linking experiments, and that a similar contact occurred with the 5' terminal portion of viral DNA, as well.

### The nucleic acid binding site of *Bacillus halodurans *RNAse H likely corresponds to the cellular DNA binding site of HIV-1 IN

Tn5 transposase is not the only IN-related protein co-crystallized in complex with a nucleic acid. Crystal structures of RNAses H in complex with RNA/DNA hybrids have been published, as well [24,25]. Of note, RNAses H are susceptible to inhibition by INSTI-related compounds [26]. To further explore possible IN/DNA interactions, a structural alignment was performed between the IN crystal structure of Goldberg *et al*. [16] and an averaged crystal structure of *Bacillus halodurans *RNAse H in complex with an RNA/DNA substrate published by Nowotny *et al*. [24]. This enzyme presents the advantage of being small and limited to the basic "RNAse H" fold, also displayed by part of the HIV-1 IN 3D architecture. The structural alignment shown in Fig. 3C involved 45 amino acids with the minimal RMSD (1.2 Å) at the level of those amino acids surrounding D71 and D132 in the RNAse H, corresponding to D64 and D116 of IN. The 3D similarities between HIV-1 IN and RNAses H have extensively been discussed in the literature [for a review, see: [27]].

When the RNA/DNA hybrid was transposed onto HIV-1 IN CCD, its projection mapped to a region within the catalytic cavity, bordering with, but distinct from the putative viral DNA-binding site, and delimited at either side by lysine residues (K136 and K159). The positive charges furnished by the metal(s) and the lysine residues are consistent with a DNA-binding region. This hypothesis is supported by structural alignments showing an overlap between a phosphate bridge of the RNA/DNA hybrid and a phosphate ion co-crystallized with HIV-1 IN by Cherepanov *et al*. [28] (Fig. 3D). Given: 1) the existence of a potential DNA-binding region adjacent to but distinct from the donor DNA-binding site in the IN catalytic site, and 2) the correspondence of this region to a well documented nucleic acid-binding site in a structurally-related enzyme (RNAse H), this region was hypothesized in the present study to be the acceptor DNA-binding site.

### Transposition of 5CITEP to a two-metal integrase model replicates contacts with flexible loop residues, Y143 and E148

To generate a surrogate platform for predicting docking of INSTIs, a model of HIV-1 IN in complex with both putative metal ions was first prepared based on homology with Rous sarcoma virus (RSV) IN. For this purpose, a structure of RSV IN CCD in complex with two metal ions [13], was superimposed to the HIV-1 IN CCD crystal structure of Maignan *et al*. Similarly to all HIV-1 IN structures in complex with metals, the structure of Maignan *et al*. presents only one metal ion in the catalytic cavity, but, differently from other published HIV-1 IN structures, displays a well ordered catalytic triad [15]. In one subunit of this structure (chain *C*), the flexible loop is present in its entirety and connects two CCD subunits in a dimer that may have biological significance, as the distance between the two active sites corresponds to 18 Å (see PDB: 1BL3), approximately one half turn of a Watson-Crick DNA helix (*i.e*., the distance at which the two antiparallel strands of acceptor DNA are simultaneously nicked during strand transfer [1,2]). The structural superimposition between the HIV-1 IN CCD and the two-metal RSV IN CCD structure involved 104 amino acids with a RMSD of 0.24 Å between the α-carbons of the highly conserved catalytic triads (D64, D121 and E157 for RSV IN). The position of the metal ion between D64 and D116 of HIV-1 IN and the metal ion between D64 and D121 of RSV IN was approximately coincident (data not shown). Then, the metal ion between residues D64 and E157 of RSV IN (corresponding to D64 and E152) was transposed onto HIV-1 IN CCD, and the E152 side chain of HIV-1 IN was moved to metal-coordinating position (matching that of the equivalent residue in RSV IN).

**Figure 4:**
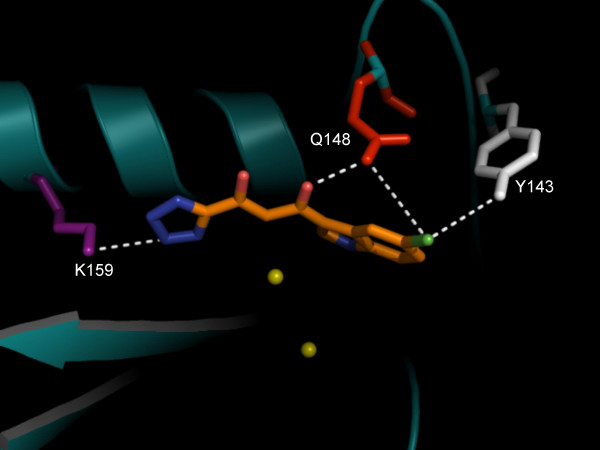
**Interaction of integrase (IN) 3' processing inhibitor, 5CITEP with HIV-1 DNA-interacting residues**. 5CITEP is shown in CPK with an orange carbon backbone. Donor DNA-interacting residues are shown in color as sticks. The putative metal ions within the IN active site are shown as yellow spheres. Possible hydrogen bonds are shown as dashed lines. Hydrogens have been removed for better clarity.

To obtain a surrogate model for a two-metal IN CCD in complex with viral DNA, the 3D coordinates of 5CITEP were extracted from the structure of Goldgur *et al*. [16] and transposed onto the two-metal model of HIV-1 IN CCD. In the newly generated complex, it was possible to place the aforementioned DNA-interacting residue Q148 at hydrogen bonding distance from the inhibitor by rotating the Cα-Cβ bond of the side chain by approx. 45° (Fig. 4). Moreover, it was possible to show an additional close contact of 5CITEP with another residue of the flexible loop, *i.e*. Y143 (not present in the 1QS4 structure), known to interact with HIV-1 DNA [28] (Fig. 4). Thus, the two-metal/IN CCD in complex with 5CITEP allowed replication of contacts occurring between HIV-1 IN and viral DNA.

### *In Silico *docking fitness of HIV-1 integrase strand transfer inhibitors (INSTIs) for the catalytic cavity of integrase in complex with 5CITEP correlates with the *in-vitro *inhibitory potencies

**Figure 5:**
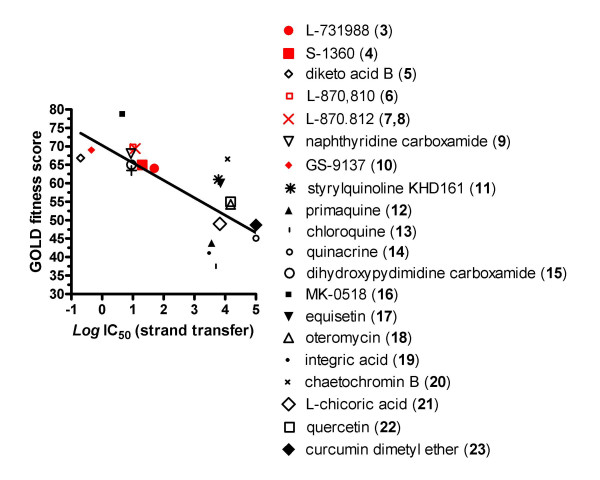
**Correlation between the inhibitory potency of different compounds on HIV-1 integrase strand transfer and *in-silico* fitness for a two-metal HIV-1 integrase core domain in complex with 5CITEP**. *x *axis: the *in-vitro *inhibitory potency of the compounds is presented as a *Log *transform of the IC_50 _value retrieved from the NCBI database (Ref. [45]). *y *axis: the *in-silico* fitness is presented as a score automatically attributed by the GOLD program. The regression line best fitting the data points is shown as a solid line. Compounds are numbered as in Fig. 2.

The two-metal/IN-CCD/5CITEP complex was used as a surrogate platform for docking simulations of IN inhibitors, *i.e*. compounds (**3–23**) (see Fig. 2). Computational simulations were conducted using the automated docking program GOLD 3.1 and the GOLD fitness function to rank the compounds on the basis of their ability to form favorable interactions. Results showed that the GOLD fitness scores of the best docking solutions correlated with the IC_50 _for strand transfer *in vitro *(*R *= -0.73; *P *= 0.001; see Fig. 5). In general, the technique allowed to distinguish between 3'P inhibitors, *e.g*. compounds (**11–13,17–22**) and selective INSTIs, *e.g*. compounds (**3–10,15,16**). The latter displayed high GOLD fitness scores (> 60). Instead, the GOLD fitness scores were lower and unrelated to the IC_50 _(*P *> 0.05) when conducted in the absence of 5CITEP, with or without the metal between D64 and E152 (data not shown).

The fitness scores obtained using the two-metal/IN-CCD/5CITEP complex are higher that those obtained by Barreca *et al*. (*i.e*., ~ 50) using the Tn5 transposase/DNA complex [19]. This is not surprising, because INSTIs were developed using HIV-1 and not Tn5-based assays [2]. On the other hand, the present study agrees with Barreca *et al*. that the acidic INSTIs have similar fitness in both the protonated and non-protonated form (data not shown).

These results allow the conclusion that occupation (by 5CITEP) of the putative donor DNA binding site is important for obtaining optimal docking of INSTIs, in line with a theory of Pommier *et al*. [1]. Moreover, the good agreement between the experimental IC_50 _values and docking solutions supports the idea that the two-metal/IN-CCD/5CITEP complex could be used as a surrogate platform for *in-silico *screening of potential INSTIs.

### Docking of integrase strand transfer inhibitors (INSTIs) reveals unexpected metal-binding modes

**Figure 6:**
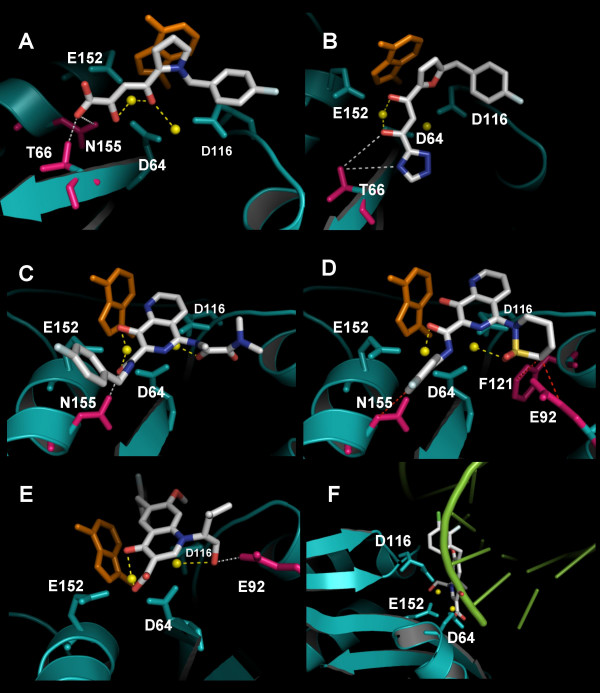
***In-silico*docking of integrase strand transfer inhibitors (INSTIs) at the integrase (IN) active site**. The structure of INSTIs L-731,988 (Panel A), S-1360 (B), L-870,812 (C), L-870,810 (D), GS-9137 (E, F) is shown in CPK. The catalytic triad (D64, D116 and E152) is shown in the same color as the protein backbone. Metal ions are presented as yellow balls. Amino acids responsible for drug resistance are colored in magenta. Significant enzyme/ligand interactions are shown as dashed lines (hydrogen bonds in white, metal coordination in yellow, Van-der-Waals forces in red). An adenine (in orange), marking the terminal portion of 3' processed viral DNA has been inserted by superimposition with the indole ring of 5CITEP. The adenine is shown for purely representative reasons, as the docking experiments were conducted in the presence of 5CITEP. A full 3D view of complexes in panels A-E can be obtained using the 3D coordinates provided as additional material [see Additional files 1, 2, 3, 4, 5,6]. In panel F, superimposition between the IN/inhibitor complex and a crystal structure of RNAse H in complex with an RNA/DNA hybrid results in an overlap between the INSTI (GS-9137 is shown as an example) and the nucleic acid (evidenced in pale green).

The docking poses of five well known INSTIs, *i.e*. compounds (**3,4,6,7/8,10**; see Fig. 2), were analyzed in further detail (the 3D coordinates of the five compounds in complex with IN CCD have been rendered available [see Additional files 1, 2, 3, 4, 5,6]). Diketo acid L-731,988, was one of the first two INSTIs to furnish proof of concept for antiretroviral effects *in-vitro *[30]. The best docking pose for this compound showed the β-hydroxy keto pharmacophore chelating both metals (Fig. 6A). Diketo-acid analog, S-1360 was the first INSTI to enter human clinical trials [1,2]. Differently from L-731,988, the functional groups of S-1360 showed, in the best docking pose, a preference for the putative metal between D64 and E152 (Fig. 6B). Both the pyrrole ring of L-731,988 and the furane ring of S-1360 showed possible π-π interactions with the indole moiety of 5CITEP. That these are false interactions artificially generated by 5CITEP is unlikely, in light of the structural similarity of the indole ring of 5CITEP and an adenine (both 5' and 3'terminal nucleotides of HIV-1 3'PDNA are adenylates) (Fig. 2 and Fig. 6B). The 8-hydroxy-1,6-naphthyridine carboxamides are an important class of INSTIs [1,2]. Naphthyridine carboxamides, L-870,812 and L-870,810 were the first INSTIs to produce proof of concept for *in-vivo *antiretroviral effects in monkeys and humans, respectively [2] A first set of docking poses (henceforth referred to as set A) had intermediate GOLD fitness scores (range: 60–65) and presented the "classic" pharmacophore described by Merck researchers (planar β-hydroxy carbonyl plus coplanar lonely-pair donor nitrogen [12]) chelating both metal ions (data not shown). Other docking poses (referred to as set B) had higher fitness scores (≅ 70; *i.e*. specifically related to potent effects on strand transfer; according to the regression line of Fig. 5). Set B indicated preferential interactions of the β-hydroxy carbonyl group with the metal between D66 and E152. Interactions consistent with coordination of the metal between D66 and D116 were present as well, but were provided by oxygens in the substituents (an acyclic amide in L-870,812, and a cyclic sulphonamide in L-870,810). Set B1, including the best ranked solution for L-870,812, displayed a β-hydroxy carbonyl that was not coplanar, due to rotation of the carboxamide group (in Fig. 6C, the acyclic amide substiturent is in *cis*, as described in Ref. [31]; see structure **8 **in Fig. 2). Rotation of the carboxamide group has been observed in other aromatic carboxamides in complex with enzymes [32,33]. Solution set B2 included the best docking solution for L-870,810 (Fig. 6D). Docking poses B1 and B2 displayed the naphthyridine rings partially overlapping on the same plane, but pose B2 slid aside from B1, thus allowing optimal positioning of the cyclic sulphonamide substituent of L-870,810 in the pocket containing the metal between D64 and D116. The docking poses of the naphthyridine carboxamides are in agreement with the novel pharmacophore described by Japan Tobaccos researchers and displayed by elvitegravir (GS-9137), a 4-quinolone 3-carboxylic acid currently in clinical trials [3,34], which does not present the lonely pair donor nitrogen coplanar to the β-hydroxy carbonyl. The best docking pose for GS-9137 presented the β-hydroxy carboxylate chelating the metal between D64 and E152 and a hydroxylic oxygen in the isobutyl substituent coordinating the other metal (Fig. 6E). In this docking solution, the carboxylate is rotated by approx. 30° from the main quinolone ring (Fig. 6E), in agreement with crystallographic data showing rotation of aromatic carboxylates in complex with metals [35].

The metal-binding mode is an unexpected finding of the present study and is a major difference with the docking results of Barreca *et al*. [19] and those of Merck researchers [12]. Both research teams described metal chelation through the "classic" pharmacophoric groups (*i.e*. a coplanar β-hydroxy keto group, to which Merck researchers add a lonely pair donor atom). Differences between the present study and that of Barreca *et al*. can of course be attributable to differences between IN and transposase. Differences with the Merck study are attributable to the fact that these authors manually drove the INSTIs into an uncomplexed IN active site [12]. It is finally possible that both docking poses A and B coexist *in vivo*, given the alternative binding modes crystallographically documented for other classes of antiretroviral drugs.

### Docking of integrase strand transfer inhibitors (INSTIs) is concordant with the drug resistance mutation profiles

To further validate the docking results, the close contacts of the INSTIs were related to well documented drug resistance mutations selected by the same inhibitors. In its best docking pose, diketo acid L-731,988 showed the carboxylate oriented towards T66, with possible hydrogen bonding (Fig. 6A). In agreement with this docking pose, T66I is a resistance mutation induced by L-731,988 which, alone, decreases diketo acid susceptibility by 6-fold [30]. Hydrogen bonding was also possible with N155, mutation of which was shown to confer cross-resistance to diketo acids [12]. S-1360, which induces drug resistance mutations similar to those selected by L-731,988 [36], also interacted with T66 (Fig. 6B). The best docking pose for L-870,812 clearly showed the carbonyl oxygen of the rotated carboxamide group directly pointing to the amide group of N155 (Fig. 6C), in perfect agreement with the drug resistance mutation N155H (*i.e*. the only known L-870,812-selected drug resistance mutation) [37]. The best docking pose for L-870,810 showed the hydrophobic portion of the sulphonamide ring in Van-der-Waals contact with the F121 sidechain (Fig. 6D), in agreement with the primary L-870,810 resistance mutation F121Y [12]. Van der Waals contacts were also possible with N155 and E92, mutations of which were shown to confer cross-resistance to this inhibitor [12,38] (Fig. 6D). The best docking pose for GS-9137 clearly presented the isobutyl substituent on the quinolone oriented towards E92 (Fig. 6E). The hydroxyl in the isobutyl substituent replaced one of the water molecules through which E92 coordinates the metal ion between D64 and E152 (see PDB structure: 1BL3 in Ref. [44]). Of note, a primary mutation induced by GS-9137 is E92Q, which, alone, is capable of decreasing drug susceptibility by 33-fold [38]. On the whole, the good agreement between the drug resistance mutation profiles and the docking poses represents a further validation of the results obtained.

### Docking of integrase strand transfer inhibitors (INSTIs) maps to the putative acceptor DNA binding site

Previous studies showing a dependence of the inhibitory activity of INSTIs from the concentration of acceptor DNA led to the hypothesis that INSTIs dock at the acceptor DNA-binding site [1,11]. If 1) this hypothesis were correct, and 2) the binding sites of INSTIs and acceptor DNA had correctly been predicted in the present study, then, structural superimpositions should result in an overlap between the docking solutions for INSTIs and the RNA/DNA hybrid in complex with *B. halodurans *RNAse H. Results showed that the docked INSTIs overlapped with the RNA/DNA hybrid when the IN/inhibitor complexes were superimposed to the *B. halodurans *RNAse H/substrate crystal structure (Fig. 6F). This result further supports the hypothesis of Pommier *et al*. that INSTIs dock at the acceptor DNA binding site of an IN/donor DNA complex [1].

### Integrase inhibitors in clinical trials are bioisosteric to fungal molecules is terms of metal binding

**Figure 7:**
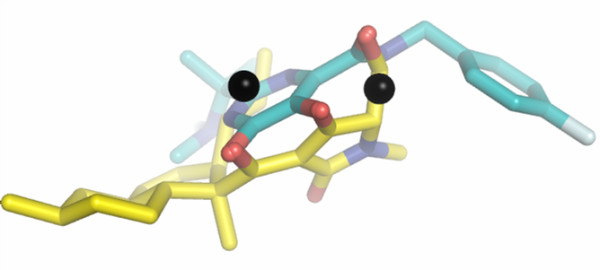
**Superimposition of the best docking solutions for natural product integrase inhibitor equisetin from *Fusarium sp*. and a 4,5-dihydroxypyrimidine carboxamide strand transfer inhibitor**. Compounds are shown in CPK. The carbon backbone of equisetin is displayed in yellow, that of the dihydroxypyrimidine carboxamide is in cyan. Metal ions are shown in black.

Interestingly, other drug classes that bind to a reaction intermediate of an enzyme/substrate DNA complex (*e.g*. topoisomerase inhibitors) are derived from natural products, thus raising the hypothesis that this type of inhibition is an enzyme inhibitory mode commonly adopted in nature and resulting from evolution within dynamic systems [1,2]. Of note, equisetin (**17**) (Fig. 2), *i.e*. the first molecule shown to inhibit strand transfer in pre-assembled IN/viral DNA complexes, was extracted from mycotoxin pools of *Fusarium sp*. [39], previously reported by Savarino *et al*. to inhibit HIV-1 integration within live cells [2,40,41]. Docking of equisetin and that of selective INSTIs were then compared. Interesting similarities were found with the best docking solution for the novel INSTI, compound (**15**), a member of the highly promising class, 4,5-dihydroxypyrimidine carboxamides/*N*-Alkyl-5-hydroxypyrimidinone carboxamides, which includes Merck's IN inhibitor raltegravir/MK-0518 (**16**) [42]. As shown in Fig.7, there is a striking overlap of the metal-coordinating groups, though not of the pending substituents. This result supports the previous idea that sesquiterpenic fungal IN inhibitors (including equisetin), though not yet acting as selective INSTIs, are different from other 3'P inhibitors. In line with this evidence, the GOLD fitness score for the interaction between equisetin and the two-metal/INCCD/5CITEP complex (*i.e*. 60) has previously been associated with enzyme inhibitory interactions and was higher than those displayed by other 3'P inhibitors such as L-chicoric acid (< 50). According to a model of Lee and Robinson, docking of L-chicoric acid requires a wide portion of the unengaged catalytic cavity [43]. The 3' P inhibitors are a heterogeneous family of molecules [2]. It is possible to speculate that some 3' P inhibitors dock at the donor DNA binding site, others occupy the entire catalytic cavity, and molecules such as equisetin can adapt to the catalytic cavity also when the donor DNA position is occupied.

## Conclusion

Molecular docking techniques may produce biologically sound results also when applied to difficult drugs targets such as an enzyme/substrate reaction intermediate. Occupation by 5CITEP of the putative donor DNA-binding site shows docking of INSTIs at a putative acceptor DNA-binding site. If future crystallographic data should confirm a similar binding of INSTIs to a IN/donor DNA complex, INSTIs might represent one of the few known drug types acting on an enzyme reaction intermediate. Moreover, novel INSTIs interact *in silico *with the metal cofactors similarly to certain natural products such as *Fusarium sp*. mycotoxins. This similarity suggests that it is possible to identify natural products drug leads capable of dissecting two different steps of an enzymatic process. The presence of potential leads for drugs of this type in natural products should encourage further natural product screening and may disclose potential drug leads targeting other nucleic acid manipulating enzymes such as the reverse transcriptase-associated RNAse H.

## Methods

### Structural alignments

3D structures were retrieved from the Protein Data Bank (PDB) [44], or from the U.S. National Center for Biotechnology Information (NCBI) website [45]. To obtain structural alignments, the α-carbons of the highly conserved catalytic triads of HIV-1 IN and related enzymes were initially superimposed using the Swiss PDB Viewer (SPDBV) program (Swiss Institute of Bioinformatics) [46], which calculates the root-mean-square distance between the corresponding atoms using a least square algorithm. Using the default matrix embedded in the program (with open and extended gap penalties of 6 and 4, respectively) [46], the calculation was extended to neighboring atoms until the maximum number of aligned atoms with the lowest RMSD was obtained. The program Pymol (v0.99; DeLano Scientific LLC, S. Francisco, CA) (freely downloadable from: [47]) was used to visualize the superimposed structures. Structural alignments were double-checked using binary *ASN1 *files and the Cn3D program (version 4.1), downloadable from the NCBI website [45].

### Generation of a two-metal/integrase model

The crystal structure of HIV-1 IN CCD solved by Maignan *et al*. [15] (PDB accession: 1BL3_C) was used as a basis for modeling the IN CCD in complex with two metal ions. Using SPDBV, this structure was superimposed to one crystal structure of RSV IN CCD (PDB: 1VSH), where two metal ions are present in the active site [13]. Using the '*torsion*' option embedded in the program, the E152 side chain was moved to metal-coordinating position (matching that of the equivalent residue in the RSV IN, E157). The position of the metal between D64 and E152 was deduced from the 3D coordinates of the corresponding metal in the aligned RSV IN.

### Molecular docking

The 3D structures of well characterized IN inhibitors including INSTIs in clinical trials were initially generated as *pdb *files using the CORINA web interface [48], on the basis of the SMILES strings published in the NCBI website [45]. The program VEGA ZZ (University of Milan, Italy; freely available at: [49]) was adopted to assign the correct bond types. The compounds were considered in their keto-enol tautomeric form, since it has been clearly established that these molecules mainly exist in this form in solution (reviewed in: [2]). Moreover, both neutral and ionic forms were generated for the carboxylic acid and triazole groups of compounds. Using the default parameters in the VEGA program, force fields and charges were assigned according to AMBER and Gasteiger algorithms, respectively, and the molecules were energy-minimized by 50 cycles of conjugate gradients (CG). Minimization was stopped when the RMSD between two subsequent solutions was lower than 0.1 Å. Energy minimized ligands were then saved as *mol *files.

A surrogate platform for molecular docking of INSTIs was generated by transposing the 3D coordinates of 5CITEP in the structure of Goldgur *et al*. [16] onto the aforementioned two-metal model of HIV-1 IN CCD, after performing a structural alignment. Water molecules were discarded from the *pdb *file, and missing side chains were reconstructed using the option '*prepare file for docking programs*' available at the WHAT-IF web interface [50]. Hydrogens were added using VEGA. The structure was then subjected to energy minimization using the default settings of the SPDBV program, *i.e*. 20 cycles of steepest descent (SD), and minimization stopping when the Δ energy was below 0.05 kJ/mol. The protein file was eventually converted to *mol2 *format using Mercury (v. 1.4.2; Cambridge Crystallographic Data Centre (CCDC); freely downloadable from: [51]). Automated docking studies were then performed using the genetic algorithm GOLD (Genetic Optimization for Ligand Docking) [52] (v. 3.1; CCDC, Cambridge, UK), according to a protocol published by Barreca *et al*. [19]. The algorithm had been previously validated and successfully tested on a data set of over 300 complexes extracted from the PDB [53]. The program was further validated in the author's hands by obtaining docking poses for HIV-1 protease inhibitors lopinavir and ritonavir nearly identical to the structures co-crystallized in complex with the HIV-1 protease (RMSD < 0.2 Å; data not shown). The binding site was initially defined as all residues of the target within 10 Å from the metal atom coordinated by D64 and D116, and later automated cavity detection was used. GOLD score was chosen as fitness function and the standard default settings were used in all calculations. For each of the 10 independent genetic algorithm runs, a default maximum of 10,000 genetic operations was performed, using the default operator weights and a population size of 100 chromosomes. Default cutoff values of 2.5 Å for hydrogen bonds and 4 Å for Van der Waals interactions were employed. The two metal ions were set to allow hexavalent coordination according to a Mg^2+ ^type (*i.e*. the metal thought to act as a co-factor *in vivo*). Carboxylate and carboxamide substituents on aromatic rings were allowed to rotate. Early termination was allowed for results differing by less than 1.5 Å in ligand all atom RMSD.

Post docking analysis was done using the program SILVER (CCDC, UK), in order to evidence close contacts such as hydrogen bonds and Van der Waals interactions.

## Competing interests

The author(s) declare that they have no competing interests.

## Authors' contributions

The author takes full responsibility for the entire content of the manuscript in that he personally conceived and designed the study, acquired and analyzed all data reported, drafted the manuscript and edited it in its final form.

## Supplementary Material

Additional file 1 **A two-metal model of HIV-1 integrase**. This file contains the 3D coordinates of the two-metal model of HIV-1 integrase described in the paper. Although the extension is *.txt*, it complies with a *.pdb *format. It can thus be opened with either programs such as MS Word or 3D molecular viewers such as the Swiss-PDB Viewer. Click here for file

Additional file 2 **L-731,988 in complex with HIV-1 integrase catalytic core domain**. This file contains the 3D coordinates of diketo acid, L-731,988, as docked at the model of HIV-1 integrase core domain in Additional file 1. Its format is the same as that of Additional file 1, and can be opened in combination with it so as to obtain a full view of this compound in complex with the two-metal model of HIV-1 integrase described in the paper. Click here for file

Additional file 3 **L-731,988 in complex with HIV-1 integrase catalytic core domain**. This file contains the 3D coordinates of diketo acid analogue, S-1360, as docked at the model of HIV-1 integrase core domain in Additional file 1. Format and instructions for opening are the same as for Additional file 2 Click here for file

Additional file 4 **L-870,810 in complex with HIV-1 integrase catalytic core domain**. This file contains the 3D coordinates of naphthyridine carboxamide, L-870,810, as docked at the model of HIV-1 integrase core domain in Additional file 1 Format and instructions for opening are the same as for Additional file 2. Click here for file

Additional file 5 **L-870,812 in complex with HIV-1 integrase catalytic core domain**. This file contains the 3D coordinates of naphthyridine carboxamide, L-870,812, as docked at the model of HIV-1 integrase core domain in Additional file 1. Format and instructions for opening are the same as for Additional file 2. Click here for file

Additional file 6 **GS-9137 in complex with HIV-1 integrase catalytic core domain**. This file contains the 3D coordinates of quinolone integrase inhibitor GS-9137, as docked at the model of HIV-1 integrase core domain in Additional file 1. Format and instructions for opening are the same as for Additional file 2. Click here for file
